# Shadow Burden of Undiagnosed Myalgic Encephalomyelitis/Chronic Fatigue Syndrome (ME/CFS) on Society: Retrospective and Prospective—In Light of COVID-19

**DOI:** 10.3390/jcm10143017

**Published:** 2021-07-06

**Authors:** Diana Araja, Uldis Berkis, Asja Lunga, Modra Murovska

**Affiliations:** 1 Department of Dosage Form Technology, Faculty of Pharmacy, Riga Stradins University, 16 Dzirciema Str, LV-1007 Riga, Latvia; 2 Institute of Microbiology and Virology, Riga Stradins University, 5 Ratsupites Str, LV-1067 Riga, Latvia; Uldis.Berkis@rsu.lv (U.B.); Modra.Murovska@rsu.lv (M.M.); 3 Development and Project Department, Riga Stradins University, 16 Dzirciema Str, LV-1007 Riga, Latvia; Asja.Lunga@rsu.lv

**Keywords:** myalgic encephalomyelitis, chronic fatigue syndrome, ME/CFS, COVID-19, diagnostic, impact on society

## Abstract

Background: Myalgic encephalomyelitis/chronic fatigue syndrome (ME/CFS) is a poorly understood, complex, multisystem disorder, with severe fatigue not alleviated by rest, and other symptoms, which lead to substantial reductions in functional activity and quality of life. Due to the unclear aetiology, treatment of patients is complicated, but one of the initial problems is the insufficient diagnostic process. The increase in the number of undiagnosed ME/CFS patients became specifically relevant in the light of the COVID-19 pandemic. The aim of this research was to investigate the issues of undiagnosed potential ME/CFS patients, with a hypothetical forecast of the expansion of post-viral CFS as a consequence of COVID-19 and its burden on society. Methods: The theoretical research was founded on the estimation of classic factors presumably affecting the diagnostic scope of ME/CFS and their ascription to Latvian circumstances, as well as a literature review to assess the potential interaction between ME/CFS and COVID-19 as a new contributing agent. The empirical study design consisted of two parts: The first part was dedicated to a comparison of the self-reported data of ME/CFS patients with those of persons experiencing symptoms similar to ME/CFS, but without a diagnosis. This part envisaged the creation of an assumption of the ME/CFS shadow burden “status quo”, not addressing the impact of COVID-19. The second part aimed to investigate data from former COVID-19 patients’ surveys on the presence of ME/CFS symptoms, 6 months after being affected by COVID-19. Descriptive and analytical statistical methods were used to analyse the obtained data. *Results:* The received data assumed that the previously obtained data on the ME/CFS prevalence of 0.8% in the Latvian population are appropriate, and the literature review reports a prevalence of 0.2–1.0% in developed countries. Regarding the reciprocity of ME/CFS and COVID-19, the literature review showed a lack of research in this field. The empirical results show quite similar self-esteem among ME/CFS patients and undiagnosed patients with longstanding disease experience, while former COVID-19 patients show a significantly lower severity of these problems. Notably, “psychological distress (anxiety)” and “episodic fatigue” are significantly predominant symptoms reported by former COVID-19 patients in comparison with ME/CFS patients and undiagnosed patients prior to the COVID-19 pandemic. The results of our analysis predict that the total amount of direct medical costs for undiagnosed patients (out-of-pocket payments) is more than EUR 15 million p.a. (in Latvia), and this may increase by at least 15% due to the consequences of COVID-19. Conclusions: ME/CFS creates a significant shadow burden on society, even considering only the direct medical costs of undiagnosed patients—the number of whom in Latvia is probably at least five times higher than the number of discerned patients. Simultaneously, COVID-19 can induce long-lasting complications and chronic conditions, such as post-viral CFS, and increase this burden. The Latvian research data assume that ME/CFS patients are not a high-risk group for COVID-19; however, COVID-19 causes ME/CFS-relevant symptoms in patients. This increases the need for monitoring of patients for even longer after recovering from COVID-19′s symptoms, in order to prevent complications and the progression of chronic diseases. In the context of further epidemiological uncertainty, and the possibility of severe post-viral consequences, preventive measures are becoming significantly more important; an integrated diagnostic approach and appropriate treatment could reduce this burden in the future.

## 1. Introduction

In recent years, the preconditions for an increase in the number of myalgic encephalomyelitis/chronic fatigue syndrome (ME/CFS) patients have emerged, and the growth rate might be contributed to by the COVID-19 pandemic. COVID-19 can induce long-lasting complications and chronic conditions such as post-viral CFS, which is a poorly understood, serious, complex, multisystem disorder, characterised by symptoms lasting at least six months, with severe incapacitating fatigue not alleviated by rest, and other symptoms—many autonomic or cognitive in nature—including profound fatigue, cognitive dysfunction, sleep disturbances, muscle pain, and post-exertional malaise, which lead to substantial reductions in functional activity and quality of life [1].

The prevalence of this disease in developed countries appears to be within the range of 0.2–1%, but this is highly dependent on case definition, geographical area, gender, and age [2]. This disease most commonly occurs between the ages of 20 and 50 years [1], thus causing a significant burden on people of working age and society as a whole. Systematic review and meta-analysis of the prevalence of (ME/CFS), performed in 2020, comprehensively estimated the prevalence of ME/CFS at 0.89%, with women approximately 1.5–2-fold higher than men in all categories. However, the prevalence rates varied widely—particularly by case definitions and diagnostic methods [3].

In Latvia, the number of patients diagnosed with ME/CFS is significantly lower than suggested by the data available in scientific literature on the prevalence of this disease. Therefore, within the framework of this study, it was planned to compare the self-reported data on observed symptoms in ME/CFS patients with those in persons experiencing symptoms similar to those of ME/CFS, but without a diagnosis. This was necessary in order to assess the likelihood and extent of latent ME/CFS in Latvia. Simultaneously, it has been hypothesised that COVID-19 might contribute to the number of undiagnosed patients with ME/CFS, and the obtained results are expected to be relevant to other countries as well.

**Figure 1:**
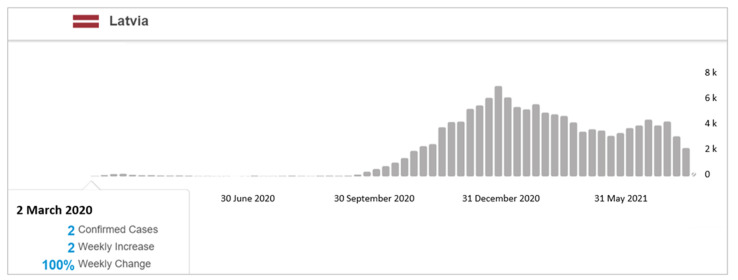
World Health Organisation Coronavirus (COVID-19) Dashboard, confirmed cases in Latvia, March 2020–May 2021 [4].

In Latvia, the first confirmed COVID-19 cases were discerned in March 2020 (Figure 1). Consequently, in the autumn of 2020, circumstances allowed for the analysis of 6 months of ME/CFS-specific exposure data for patients affected by COVID-19 in March 2020.

The number of patients affected by COVID-19 was relatively small in March 2020, and this allowed us to develop a high-coverage cohort to conduct the study. Additionally, a literature review was performed to compare the data obtained in this empirical study with data from other studies. The literature review was devoted to the classic factors assumedly affecting the diagnostic scope of ME/CFS, and the causal interaction between ME/CFS and COVID-19 as a new contributing factor.

Consequently, the aim of this research was to investigate the issues of potential undiagnosed ME/CFS patients in Latvia, with a hypothetical forecast of the expansion of a post-viral CFS as a consequence of COVID-19 and its burden on society. The burden of undiagnosed ME/CFS can be described as a shadow burden. To achieve the aim of this research, the following tasks were defined:

- Estimate the literature on classic factors presumably affecting the diagnostic scope of ME/CFS, and their ascription to Latvian circumstances, as well as conducting a literature review to assess the potential relationship between ME/CFS and COVID-19 as a new contributing agent, and its reflection in scientific literature;
- Analyse data from the survey performed both for ME/CFS patients and for persons experiencing symptoms similar to those of ME/CFS, but without a diagnosis (prior to the COVID-19 pandemic), in order to compare the certain socioeconomic and disease management aspects for patients and potential undiagnosed patients. Data from the ME/CFS patients’ survey were previously analysed in a comparative study with Italy and the UK. Conversely, the data from undiagnosed patients were not analysed previously; nevertheless, these data create significant potential for assessing the shadow impact of ME/CFS;
- Test the possible interaction between COVID-19 and ME/CFS in Latvian circumstances, by conducting a survey of former COVID-19 patients on the presence of ME/CFS symptoms;
- Make preliminary predictions on the potential shadow impact of ME/CFS on society, limiting this study to direct costs for patients.

The first section of this article is devoted to theoretical aspects and literature review, followed by the description of the methods and materials used in the empirical research, and the presentation of the results. The discussion section draws attention to the potential impact of ME/CFS on society in the light of COVID-19. The publication is finalised by conclusions and recommendations for further research.

## 2. Theoretical Background and Literature Review

Theoretical contemplations are elaborated in this section, with the initial focus on classic factors assumedly affecting the diagnostic scope of ME/CFS, and their ascription to Latvian circumstances. The classification of diagnoses is one of these factors, and the World Health Organisation’s approach is used for these purposes in Latvia. To classify ME/CFS by the World Health Organisation’s International Statistical Classification of Diseases and Related Health Problems (ICD-10), mainly, two ICD-10 codes—code G93.3 (post-viral fatigue syndrome/myalgic encephalomyelitis (ME)) and code 52.82 (chronic fatigue syndrome (CFS))—are used [5]. Myalgic encephalomyelitis (ME), identified as a new clinical entity with distinctive features in 1956, was originally considered to be a neuromuscular disease [6]. In turn, several case definitions were developed in order to improve the comparability and reproducibility of clinical research and epidemiologic studies. Since the first ”ME” case definition was developed in 1986, 25 case definitions/diagnostic criteria were created based on three conceptual factors (aetiology, pathophysiology, and exclusionary disorders). These factors can be categorized into four categories (ME, ME/CFS, CFS, and SEID (systemic exertion intolerance disorder)) [7].

There are eight most prominently cited case definitions and diagnostic criteria, which can be applied for each of the following categories:

- CFS (Fukuda et al. (US Centre for Disease Control (CDC, 1994)) [8], Holmes et al. (1988) [9], Australian (1990) [10], Oxford (1991) [11]);
- ME (Ramsay et al. (1992) [12] and International Consensus Criteria (ICC, 2011) [13]);
- ME/CFS (Canadian Consensus Criteria (CCC, 2003) [14]), and;
- SEID (IOM, 2015) [15], according to the focus of the primary disorder [7].

SEID was proposed by the Institute of Medicine (IOM, now the National Academies of Medicine (NAM), Washington, DC, USA) to resolve diagnostic confusion, as a new clinical entity to replace ”ME/CFS”. SEID is defined by chronic fatigue, post-exertional “malaise”, and unrefreshing sleep, as well as orthostatic intolerance and/or cognitive impairment [16]. However, SEID case criteria do not do justice to either ME or CFS, nor to their definitions. Furthermore, in addition to the theoretical impossibility of replacing two different definitions with a new definition, the SEID case criteria are also applicable to subsets of people with other diseases—for example, multiple sclerosis (MS) and lupus—and psychological conditions—for example, major depression—while only a subset of people with the diagnosis of CFS meet the diagnosis of SEID.

The introduction of SEID did not resolve the impasse, but highlighted the uncertainties of the diagnoses and the need to seek new approaches to improve the diagnostic process.

The authors of this article assume that the discovery of biomarkers and the use of machine learning capacities are the most state-of-the-art approaches to improve the diagnostic process. Several original studies and literature reviews demonstrate the potential of biomarkers in the diagnosis of ME/CFS, and the contribution of precision medicine and personalised healthcare [17,18,19]. The European ME/CFS Research Network (EUROMENE) (in which Latvia is represented by the Riga Stradins University) has established a database for active biomarker research in Europe, called the EUROMENE ME/CFS Biomarker Landscape project [20]. In Latvia, the investigation of ME/CFS biomarkers is also encouraged and supported by the Latvian Science Council’s Fundamental and Applied Research project No lzp-2019/1-0380 “selection of biomarkers in ME/CFS for patient stratification and treatment surveillance/optimisation”.

In turn, artificial intelligence and machine learning can greatly support the diagnostic process; however, problems with the initial identification of patients remain topical. In this process, general practitioners (GPs) have an important role, and EUROMENE participants performed a literature review of GPs’ knowledge and understanding of ME/CFS (papers were mostly from the United Kingdom), concluding that disbelief and lack of knowledge and understanding of ME/CFS among GPs is widespread, and the resultant diagnostic delays constitute a risk factor for severe and prolonged disease. Failure to diagnose ME/CFS renders attempts to determine its prevalence and, hence, its economic impact, problematic [21]. In addition, a survey of academic and clinical experts who are participants in EUROMENE was conducted to elicit perceptions of GPs knowledge and understanding of ME/CFS, and the results of this survey reported that lack of knowledge and understanding of ME/CFS among GPs is a major cause of missed and delayed diagnoses, which renders attempts to determine the incidence and prevalence of the disease, and to measure its economic impact, problematic. It also contributes to the burden of disease through mismanagement in its early stages [22]. A comparative survey of people with ME/CFS in Italy, Latvia, and the United Kingdom, performed on behalf of the Socioeconomics Working Group of the EUROMENE, indicated that GPs more frequently had principal responsibility for medical care in Latvia than in Italy or the UK, and this probably reflects the fact that in Latvia GPs perform the gatekeeper role for patients in the diagnostic and treatment process [23].

An additional determining factor is the patients’ engagement in outcome measurement and disease management. A literature review performed 10 years ago drew conclusions that the quality and acceptability of reviewed patient-reported outcome measures (PROMs) were limited, and recommendations for patient-reported assessment were difficult [24]. Clear discrepancies existed between what was measured in research and how patients defined their experience of ME/CFS. It was recommended that future PROM development/evaluation must seek to involve patients more collaboratively, in order to measure outcomes of importance using relevant and credible methods of assessment [24]. 10 years later, the situation is more comprehensive, and one literature review defines in total 15 patient-reported outcome (PRO)-derived tools (used in 50 randomised clinical trials (RCTs)) along with two behavioural measurements for adolescents (4 RCTs). The review comprehensively provides the choice pattern of the assessment tools for interventions in RCTs for ME/CFS [25]. However, the environment of RCTs is different from the environment in which patients live daily.

Taking into account the identified challenges that accompanied the process of collecting PROs in the daily lives of ME/CFS patients, EUROMENE member countries’ representatives have defined a view on the creation of an app and a web platform for ME/CFS patients’ self-empowerment and disease management, where the target users are people suffering from ME/CFS, and the practical challenge is diagnosis, stratification, and monitoring of ME/CFS at the level of the GP, supported by the virtual doctors’ consortium, as well as patient self-awareness and proper practical navigation in the healthcare system [26]. This project is currently in the process of seeking funding.

At the same time, the COVID-19 pandemic, resulting from severe acute respiratory syndrome coronavirus 2 (SARS-CoV-2), has severely impacted the population worldwide, with a great mortality rate. According to the lessons from past epidemics, previous research on post-epidemic and post-infection recovery has suggested that the complications include the development of severe fatigue. Certain factors, such as the severity of infection, in addition to the “cytokine storm” experienced by many COVID-19 patients, may contribute to the development of later health problems [27].

In the light of COVID-19′s epidemiological uncertainty, the issues of the causes and consequences of the disease remain topical, and CFS is a possible predictor and consequence of COVID-19. A literature review, which included 1161 primary studies published between January 1979 and June 2019, concluded that the four most common causal factors of ME/CFS were: immunological (297 studies), psychological (243), infections (198), and neuroendocrine (198) [28]. The causes can be broadly characterized according to primary disorder (ME—viral, CFS—unknown, ME/CFS—inflammatory, SEID—multisystemic), compulsory symptoms (ME and ME/ CFS—neuroinflammatory, CFS and SEID—fatigue and/or malaise), and required conditions (ME—infective agent, ME/CFS, CFS, SEID—symptoms associated with fatigue, e.g., duration of illness) [7].

**Figure 2:**
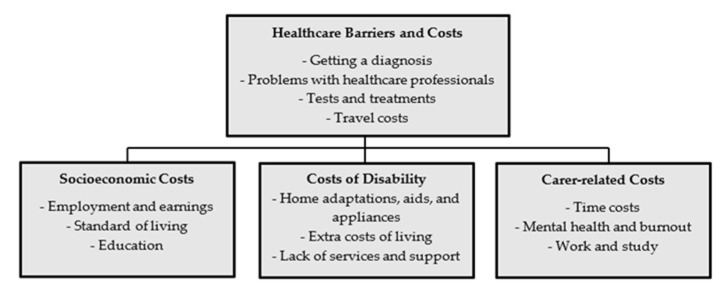
Economic impact of myalgic encephalomyelitis/chronic fatigue syndrome (ME/CFS) [29].

Therefore, the increase in the number of undiagnosed ME/CFS patients is becoming specifically relevant in the light of the COVID-19 pandemic. Theoretically, the economic impact assessment of this disease could be based on the current level of costs (direct, indirect, and intangible) to society, by modelling and forecasting techniques. However, data on the prevalence of ME/CFS are widely dispersed, and data on financial impact are even more uncertain. In the framework of EUROMENE, representatives of Ireland in the Socioeconomic Working Group have performed a qualitative study on understanding the economic impact of ME/CFS in Ireland [29]. The identified healthcare barriers and costs are described in Figure 2.

Participants in the mentioned study described a range of problems and costs that related to getting a diagnosis of ME/CFS. As described in the study, for some it took years, with numerous visits to GPs, consultants, and other healthcare professionals, for their illness to be identified or even acknowledged. Participants highlighted how they were often passed from one healthcare professional to another. In many cases, consultations to get a diagnosis were paid for out-of-pocket, at significant personal cost [29].

**Figure 3:**
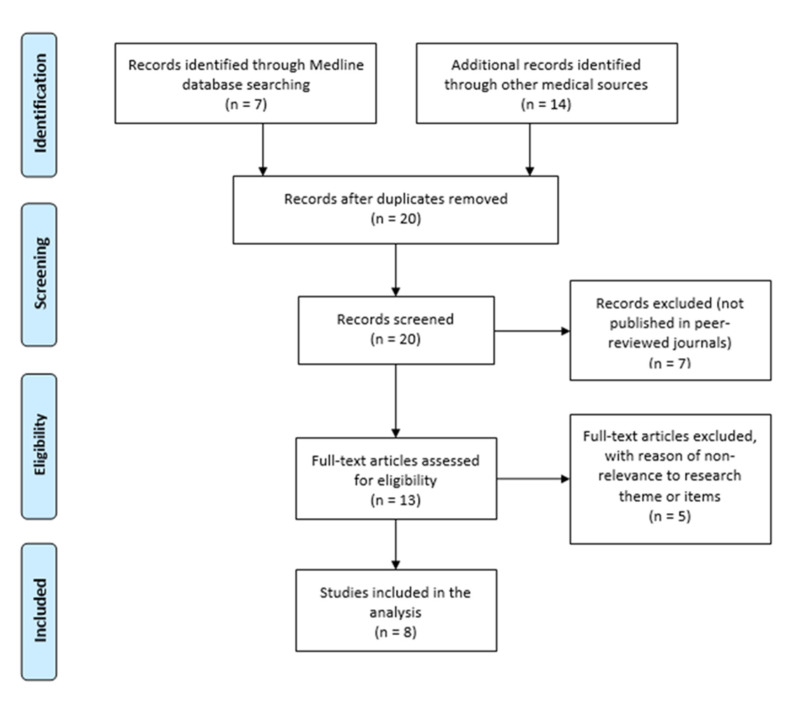
Flow diagram of the selection process for the literature review on the possible interaction between ME/CFS and COVID-19.

In the theoretical research on the causal interaction between ME/CFS and COVID-19, the purpose was to identify the main findings regarding the reciprocity of ME/CFS and COVID-19. The search was performed on Medline (via PubMed) and other relevant scientific databases (without restriction for publishing period). The following search key words were used: (“COVID-19”) OR (“coronavirus”) OR (“SARS-COV-2”) AND (“chronic fatigue syndrome”) OR (“myalgic encephalomyelitis”) OR (“CFS”) OR (“ME/CFS”). The flow diagram of the selection process is shown in Figure 3.

A total of 21 articles were identified using the aforementioned search strategy (Figure 3). After the removal of duplicates using reference management software (EndNote, Clarivate Analytics), 20 articles were screened for title and abstract, and 7 articles were excluded due to not being published in peer-reviewed journals. The remaining 13 articles were screened against eligibility criteria; 5 full-text articles were excluded for non-relevance to the research theme or items, and therefore 8 articles were included in the analysis.

The main findings of the literature review are presented in a summary of findings table (Table 1); this table provides key information concerning the research’s authors, type of research, and the sum of available data on the main outcomes.

The publication period for identified scientific literature was not defined, but the first relevant publication was dated to October 2020, and more research articles were published in 2021. Table 1 shows the main research outcomes of published scientific literature in peer-reviewed journals. Note that the authors pay attention not only to the symptoms, but also to changes in quality-of-life indicators.

## 3. Materials and Methods of Empirical Research

**Figure 4:**
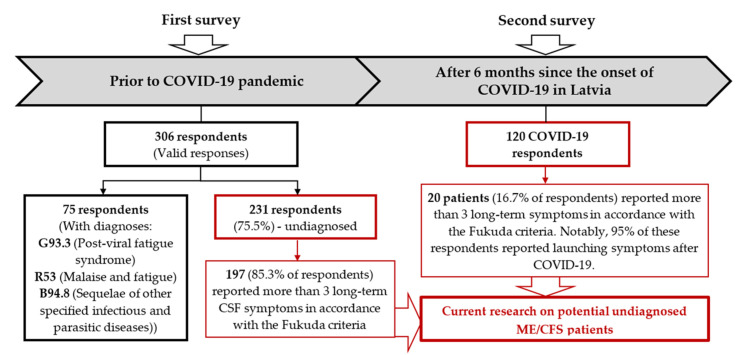
The empirical study design and outcomes.

This section is devoted to the empirical research conducted by the authors to investigate the shadow burden of ME/CFS and its causal interaction with COVID-19 in the context of Latvia. To achieve the aim and objectives of the empirical research, the study design consisted of two parts (Figure 4):

(1) The first part was dedicated to comparison of self-reported data from ME/CFS patients with those from persons experiencing symptoms similar to those of ME/CFS, but without a diagnosis, obtained by the survey performed prior to the COVID-19 pandemic. This part envisaged the creation of an assumption on the ME/CFS shadow burden “status quo”—not addressing the impact of COVID-19—in Latvia.
(2) The second part aimed to investigate the data from COVID-19 patients’ surveyed on the presence of ME/CFS symptoms, 6 months after being affected by COVID-19, in Latvia.

The first patients’ survey (Data S1) was designed mostly to obtain general information (e.g., age, gender, education, etc.) and information on their symptoms, clinical history, and the socio-economic consequences of the disease—including restrictions on daily life, sources of assistance, and understanding and awareness of the disease. The purpose of the survey was indicated in the introductory part of the questionnaire—to evaluate patients’ knowledge about ME/CFS, health care received, and problems related to the impact of ME/CFS on quality of life. The questionnaire was addressed to persons who experienced chronic fatigue for at least six months that could not be reduced by rest, headache, muscle aches, enlarged lymph nodes, joint pain, neck pain, memory problems, sleep problems, and other typical symptoms. The survey was approved by the Research Ethics Committee of the Riga Stradins University (Decision No. 6-3/3, 25 October 2018, Riga), launched in February 2019, and lasted for two months. The survey was distributed through GPs, as well as on the social networking platform Mammamuntetiem.lv (accessed on 18 February 2019; a portal for families and parents) that was most relevant to the structure of potential patients (mostly used by persons between the ages of 20 and 50 years). A total of 306 valid responses were received, of which 75 were from patients with G93.3, R53, and B94.8 diagnoses, while 231 respondents had reported CFS-like symptoms but had not been diagnosed (Figure 2). The results of diagnosed ME/CFS patients’ surveys were investigated in the scope of Brenna et al.’s Comparative Survey of People with ME/CFS in Italy, Latvia, and the UK [23]. At the same time, the data of undiagnosed patients were not properly analysed, and in this study the authors emphasise the issues of undiagnosed patients, and the possible increase in their number due to the COVID-19 pandemic.

Therefore, the second survey was dedicated to potential ME/CFS patients in the post-COVID-19 phase. This survey’s data were obtained from a cohort of former COVID-19 patients established at the Genome Database of Latvian Population national biobank [38], in accordance with the Central Medical Ethics Committee’s (Latvia) approval No 01-29.1/5034 (23 September 2020, Riga). ME/CFS was a secondary objective of this questionnaire; therefore, data for the present study were limited to questions about the presence of ME/CFS-like symptoms and quality of life. In Latvia, the first confirmed COVID-19 cases were discerned in March 2020, and consequently, the former COVID-19 patients affected in March 2020 were surveyed in October and November 2020, to establish a 6-month ME/CFS-specific exposure period.

Both questionnaires (inter alia) contained questions about CFS-relevant symptoms, in accordance with the CDC-1994 (Fukuda) criteria. The CDS-1994 case definition and criteria were chosen, as EUROMENE suggests mostly using the Fukuda definition and CCC definition, which identify a more severely affected group of patients. The CDC-1994 definition appeared more robust and less likely to be affected by variations in data collection methods [1]. The threshold was defined as four required accompanying symptoms, in accordance with Fukuda et al. [8,39]. Additionally, quality-of-life measurement was performed. Patients were asked to rate their quality of life (QoL) on a scale from 0 to 100 (where 100 represents the best possible QoL, and 0 the worst) for the year prior to onset of illness, and for the year immediately preceding completion of the survey. The current level of health-related quality of life was assessed using the EuroQol-5D-5L measure (certified translation: EQ-5D-5L Latvian for Latvia). Descriptive and analytical statistical methods were utilised for analysis of the obtained data.

In the Discussion section, the statistical data provided by the national competent authorities of Latvia were also used to make preliminary predictions about the potential shadow impact of ME/CFS on society.

## 4. Results

This section presents the outcomes of two surveys according to the research methodology: The first—a “status quo” survey—compares data from two groups of respondents prior to the COVID-19 pandemic: self-reported data from ME/CFS patients, and from persons experiencing symptoms similar to those of ME/CFS, but without a diagnosis. The second survey presents former COVID-19 patients’ data in order to analyse the presence of ME/CFS-like symptoms and predict ME/CFS expansion.

The main data of descriptive statistics of the first survey are shown in Table 2; There were 75 valid responses from ME/CFS patients, consisting of 62 women and 13 men (20 patients with G93.3 disease code, 46 patients with R53 disease code, and 11 patients with B94.8 disease code; two patients had double diagnoses). Concerning the potentially undiagnosed patients, there were 231 completed responses (with different participation in completing certain questions) but, in both groups, the proportion of females was the same—82.7%. The patients’ average age was 50 years (the respondents ranged in age from 17 to 81), while for undiagnosed persons it was 45 years. Other sociodemographic information shows that 60% of patients were married; in both groups, around of a third of respondents lived alone. In addition, 43% of patients were graduates, but a higher proportion (more than half) of undiagnosed persons with higher education degrees was observed. Additionally, comparative results are presented in Table 2 under the following items—household income (by household member), out-of-pocket payments to mitigate the consequences of illness and syndrome, number and variability of symptoms, number of investigations, difficulty explaining the illness and syndrome, and quality of life.

It is assumed that this disease has significant impacts on personal income, because patients are frequently unable to work, and spend out-of-pocket resources for treatment. In order to assess the financial situation of patients in Latvia, the data of the Central Statistical Bureau on the mean disposable (net) income per household member were used for comparison. In Latvia, the mean disposable (net) income per household member in 2019 was EUR 6994 [40]. In accordance with the survey data, 48 of the ME/CFS patients (73.9%) reported lower than mean net income per household member, but still, on average, spent more than EUR 1140 p.a. on symptom relief. In the group of undiagnosed persons, 141 respondents (66.2%) reported lower than mean net income per household member, with a slightly lower out-of-pocket payment of EUR 979 p.a. for the mitigation of symptoms and their consequences.

Patients presented on average 7–8 different symptoms, and 9% of patients presented more than 10 symptoms. Significantly, undiagnosed persons reported more than 6 symptoms on average, and 197 (85.3%) of 231 respondents reported more than 3 long-term symptoms similar to ME/CFS symptoms, which is the threshold for the Fukuda criteria. The number of investigations prior to reaching a diagnosis on average was 6, and 43% of patients indicated that more than 12 months passed from their first symptoms to reaching a diagnosis. There was no significant difference in the number of investigations between patients and undiagnosed persons, but for the last group, it had not resulted in reaching a diagnosis. Both groups indicated high variability in symptoms (more than 70%), and undiagnosed persons were more likely to describe their symptoms as variable. The difficulty in explaining the symptoms can be one of the major difficulties for ME/CFS patients (almost 27% of patients reported difficulties in explaining their symptoms to physicians, 47% to family members, 27% to friends, and 40% to employers). The most critical point for both groups is explanation of their symptoms to family and employers.

Concerning the effectiveness of therapies, 64% patients noted the effectiveness of medication (prescription and OTC medicines), and 52% patients reported the effectiveness of non-medication methods (physiotherapy, psychotherapy, osteopathy, homeopathy, nutrition, and food supplements) and complex methods. The complex approach probably provides additional benefits of treatment, taking into account the multisymptom nature and aetiology of the condition. No caregivers other than family were reported; patients mostly took care of themselves.

In the empirical part of the research on investigation of the interaction between ME/CFS and COVID-19, the former COVID-19 patients’ survey (the second survey) was performed as a part of the project on the evaluation of the data of the cohort of former COVID-19 patients established at the Genome Database of Latvian Population national biobank. Taking into account that the first confirmed cases of COVID-19 were discerned in March 2020, a 6-month period was required to obtain ME/CFS data, and former COVID-19 patients—affected by COVID-19 in March 2020—were surveyed in October and November 2020. In March 2020, there were 204 confirmed cases of COVID-19 in Latvia [4]. Subsequently, the patients who were affected by COVID-19 in March 2020 were invited to volunteer for a telephone survey in October–November 2020. Respectfully, 120 people agreed, and responded to questions on ME/CFS symptoms and health-related quality of life. Consequently, the sample covers more than half of the patients infected in March 2020, and the data obtained are statistically significant.

**Figure 5:**
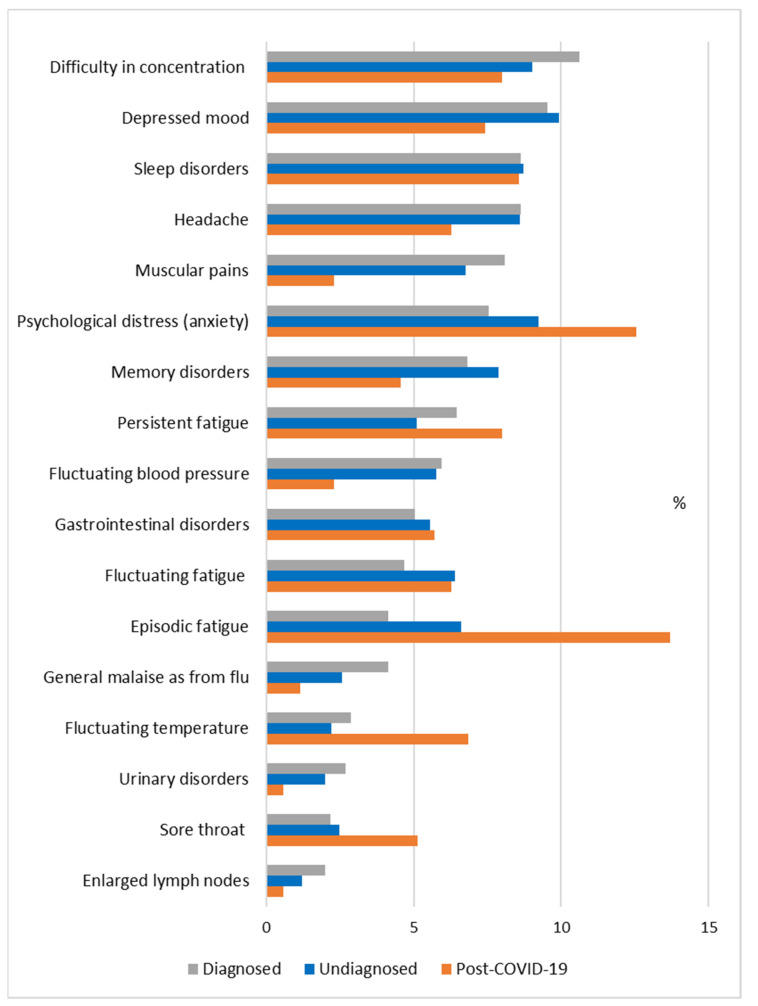
The prevalence of symptoms reported by ME/CFS patients and undiagnosed patients prior to COVID-19 (the first survey), and by former COVID-19 patients 6 months after infection (the second survey), as a percentage of the total number of reported cases of symptoms in each group.

The data of the survey showed that 53 patients (44.2%) out of 120 respondents who had not been diagnosed with ME/CFS prior to the COVID-19 pandemic reported at least one of the symptoms characteristic of CFS, in accordance with the Fukuda criteria; 20 respondents (16.7%) reported 4 or more CFS-specific symptoms simultaneously. In order to compare the dominance of symptoms occurring in former COVID-19 patients with data from ME/CFS patients and undiagnosed patients prior to COVID-19, the relevant data are summarised in Figure 5.

The data show (Figure 5) that the predominant symptom in ME/CFS patients is “difficulty concentrating”, while in undiagnosed patients “depressed mood” predominates, and “sleep disorders” puts an equally hard burden on all groups of patients. Significantly, non-diagnosed patients and former COVID-19 patients have noticeably higher levels of “psychological distress (anxiety)” compared to ME/CFS patients. “Muscular pain” and “headache” are vastly less common in former COVID-19 patients, but “sore throat” is substantial. “Memory disorders” occur more in undiagnosed patients. “Fluctuating blood pressure”, “general malaise, as from flu”, “urinary disorders”, and “enlarged lymph nodes” are more common in ME/CFS patients. Regarding the different manifestations of fatigue, it should be noted that “persistent fatigue” is more common in former COVID-19 patients and ME/CFS patients, whereas “fluctuating fatigue” is more common in undiagnosed patients. “Episodic fatigue” is relatively less common in ME/CFS patients, but is predominant in former COVID-19 patients and undiagnosed patients. Former COVID-19 patients are also characterised by “fluctuating temperature” and slightly greater dominance of “gastrointestinal disorders” compared to ME/CFS patients and undiagnosed patients.

Noticeably, 95% of the post-COVID-19 respondents reported onset of symptoms after being affected by COVID-19. This allows the assumption of COVID-19 as a causative agent of CFS, and probably of ME/CFS.

**Figure 6:**
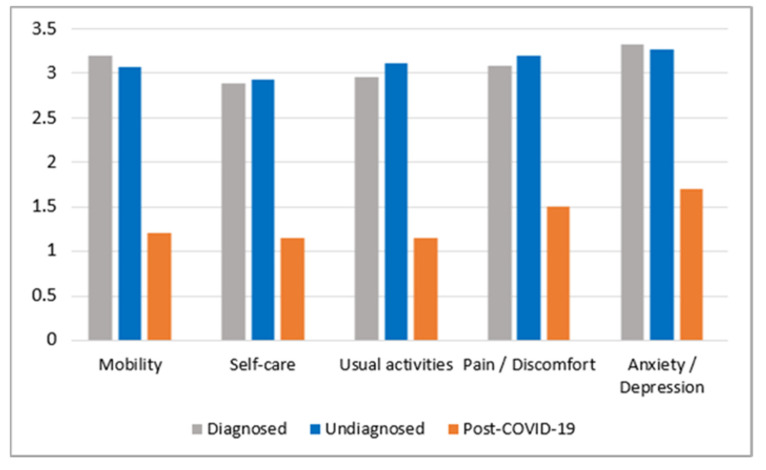
Patient-reported health-related quality of life, as measured by the EuroQol-5D-5L framework (1—the best possible option, and 5—the worst), in ME/CFS patients and undiagnosed persons prior to COVID-19, and former COVID-19 patients (6 months after being affected).

Concerning to the health-related quality of life measurements, the EuroQol-5D-5L was used to analyse the patients’ self-esteem in the following fields: mobility (no/problems in walking about), self-care (no/problems washing or dressing myself), usual activities (e.g., work, study, housework, family or leisure activities—no/problems doing usual activities), pain/discomfort (no/pain or discomfort), and anxiety/depression (not/anxious or depressed). The results are summarised in Figure 6.

Data on ME/CFS patients (74 respondents), as well as undiagnosed patients (196 respondents) and former COVID-19 patients (20 respondents) who reported four or more ME/CFS-like symptoms, were used to obtain comparable data. The results show (Figure 6) quite similar self-esteem among ME/CFS patients and undiagnosed patients with long-standing disease experience, while former COVID-19 patients show a significantly lower severity of these problems.

It is important to note that there is no considerable difference in self-reported quality of life (using the VAS) between the ME/CFS patients group and the undiagnosed persons group (Table 2) prior to illness and in the past year. Significantly, the quality of life prior to illness was relatively low (scoring less than 75 out of 100), considering the average age of the target groups, and this encourages deeper research in the context of the overall quality of life of the Latvian population.

## 5. Discussion

As mentioned in the Materials and Methods section, this section is complimented with statistical data provided by the national competent authorities of Latvia, so as to contribute preliminary predictions about the potential shadow impact of ME/CFS on society.

The previously analysed data from the Latvian Centre for Disease Prevention and Control (CDPC) and the National Health Service (NHS) of Latvia tentatively indicated high prevalence of ME/CFS in Latvia. CDCP data from primary care indicated that approximately 700 patients had ICD-10 code G93.3 assigned, while there were approximately 15,000 with ICD-10 code R53, and about 70 with code B94.8. In total, these constitute about 0.8% of the Latvian population, which is considerably higher than the prevalence found in other comparable populations [1]. When discussing these data within the EUROMENE network, the prevalence seemed too high. However, an analysis of the literature shows that there are still no clear definitions of the exact classification of related diseases and case definitions. In addition, new approaches, new disease designations, and a nomenclature of syndrome sets are emerging. GPs, on the other hand, point to problems in making a precise diagnosis [21,22,23]. In these circumstances, it is possible that the obtained data on the prevalence of 0.8% in Latvia are appropriate, taking into account the fact that the literature review reports a prevalence of 0.2–1.0% in developed countries [2].

In accordance with the data from the Central Statistical Bureau of Latvia, at the beginning of 2020 the population of Latvia was approximately 1,908,000 people [41]. Accordingly, the prevalence of the disease may vary from 3816 to 19,080 patients in Latvia. In 2019, the NHS data show that 3142 patients of diagnosis codes G93.3 (post-viral fatigue syndrome), R53 (malaise and fatigue), and B94.8 (sequelae of other specified infectious and parasitic diseases) received the treatment from the state budget. The survey of potential ME/CFS patients performed in the scope of this research shows that undiagnosed persons reported more than 6 symptoms on average, and 197 (85.3%) of 231 respondents reported more than 3 long-term CFS-like symptoms, which is the threshold for the Fukuda criteria. At the same time, there was no significant difference in self-reported quality of life between the patients group and the undiagnosed persons group. These data confirm a high level of undiagnosed patients in Latvia.

Regarding the correlation with COVID-19, it could be generally assumed that post-viral fatigue syndrome (myalgic encephalomyelitis (ME)) is a logical consequence of viral infection. However, concerning CFS there are currently insufficient data to statistically confirm or reject this interaction. The literature review revealed a lack of research in this field. The present research indicates that the number of undiagnosed ME/CFS patients might increase by at least 15% due to the COVID-19 pandemic. In these circumstances, the COVID-19 pandemic presents a new potential challenge to increase the shadow burden of ME/CFS. The survey data of Latvian COVID-19 patients report alarming results for CFS-like symptoms after COVID-19 infection. Particular attention should be paid to the fact that “psychological distress (anxiety)” and “episodic fatigue” are significantly prevalent symptoms reported by former COVID-19 patients, in comparison with ME/CFS patients and undiagnosed patients prior to the COVID-19 pandemic. Health-related quality-of-life measurements according to EuroQol-5D-5L show better results in former COVID-19 patients compared to ME/CFS patients and undiagnosed patients prior to the COVID-19 period, but this may be explained by the relatively short time period in which persistent symptoms could be observed in former COVID-19 patients.

Concerning the shadow financial burden of ME/CFS on society, with respect to the direct costs faced by potential patients, the survey’s data can be useful to predict the approximate out-of-pocket treatment cost per patient. In accordance with the survey data, 73.9% of the ME/CFS patients reported lower than mean net income per household member, but still, on average, spent more than EUR 1140 p.a. on symptom relief. In the group of undiagnosed persons, 66.2% of respondents reported lower than mean net income per household member, with a slightly lower out-of-pocket payment of EUR 979 p.a. for the mitigation of disease consequences.

Assuming that the actual number of patients in Latvia is—for instance—15,770 patients, as forecasted by the CDPC, and each of them spend EUR 979 p.a. to reduce the consequences of the disease, the total direct medical cost for undiagnosed patients is more than EUR 15 million p.a., and may increase by at least 15% in response to the influence of COVID-19. 

In these circumstances, prevention programmes can play a significant role, and provide economic benefits as primary prevention and secondary prevention to minimise the diagnostic delays associated with prolonged illness, increased severity, and increased costs [42]. Data on quality of life are also noteworthy, as quality of life prior to illness, as reported by the survey, was relatively low (scoring less than 75 out of 100) considering the average age of the target groups (45–50 years old), and this encourages deeper research in the context of the overall quality of life of the Latvian population.

The present research creates the foundation for determining the “status quo” of undiagnosed patients with ME/CFS in Latvia, and propounds a vision for the further development of the scenario in the light of COVID-19. Simultaneously, the study has several limitations, the most substantial of which is related to the cohort formation of former COVID-19 patients, taking into account that a 6-month period is required to assess the presence of ME/CFS symptoms. The most significant number of confirmed COVID-19 cases in Latvia was observed in December 2020 (Figure 1); thus, in the second half of 2021 it would be valuable to continue this study, with a larger coverage of patients.

## 6. Conclusions

We came to the realisation that ME/CFS creates a significant shadow burden on society, even taking into account only the direct medical costs of undiagnosed patients—the number of whom in Latvia is probably at least five times higher than the number of discerned patients. A similar situation can be observed in other countries. Simultaneously, the hypothesis tends to be confirmed that COVID-19 might contribute to the number of undiagnosed patients with ME/CFS, and COVID-19 can induce long-lasting complications and chronic conditions—such as post-viral CFS—and increase this burden. The Latvian research data assume that ME/CFS patients are not part of the high-risk group for COVID-19; however, COVID-19 causes ME/CFS-relevant symptoms in patients. This increases the need for monitoring of patients for even longer after recovering from COVID-19′s symptoms, in order to prevent complications and the progression of chronic diseases, including ME/CFS. In the context of further epidemiological uncertainty and the possibility of severe post-viral consequences, preventive measures are becoming significantly important, as well as an integrated use of the criteria, identification of biomarkers, and the aid of artificial intelligence for diagnostic purposes and appropriate treatment, all of which could help to reduce this burden in the future. The increased risk of worse outcomes from COVID-19 should be taken into account in decision-making with regard to individual and population-wide risks, prevention, and detection measures.

## Figures and Tables

**Table 1:** Characteristics of the scientific articles included in our analysis to assess the possible interaction between ME/CFS and COVID-19.

Authors	Type of Research	Main Results and Conclusions
Strayer et al. (Oct 2020) [30]	Research Article	The results may have direct relevance to the cognitive impairment and fatigue being experienced by patients clinically recovered from COVID-19 and free of detectable SARS-CoV-2.
Gaber (Jan 2021) [31]	Review	Post-viral fatigue is the most common long-term health issue facing survivors of COVID-19, according to initial reports. The author discusses the risk, diagnosis, and principles of management of post-viral fatigue and its chronic variant—ME/CFS—within the context of the pandemic, and highlights that further research is urgently needed to guide clinical practice. Several symptoms are classically associated with post-viral fatigue and ME/CFS, including physical pain, recurrent headaches, malaise, cognitive impairment, unrefreshing sleep, recurrent sore throats, and lymphadenopathy. These symptoms are strongly associated with the post-exertional phase of the boom-and-bust cycle. Identification of the post-COVID patients needing support and treatment should be a part of the overall COVID-19 response globally.
Friedman et al. (Feb 2021) [32]	Opinion	The similarity and overlap of ME/CFS and long-haul COVID-19 symptoms suggest similar pathological processes. A unifying hypothesis explains the precipitating events, such as viral triggers and other documented exposures; for their overlap in symptoms, ME/CFS and long-haul COVID-19 should be described as post-active-phase-of-infection syndromes (PAPISs). The authors further propose that the underlying biochemical pathways and pathophysiological processes of similar symptoms are similar regardless of the initiating trigger. The authors caution that failure to meet the now combined challenges of ME/CFS and long-haul COVID-19 will impose serious socioeconomic as well as clinical consequences for patients, the families of patients, and society as a whole.
Halpin et al. (Feb 2021) [33]	Research Article	There is currently very limited information on the nature and prevalence of post-COVID-19 symptoms after hospital discharge. In this research, a purposive sample of 100 survivors discharged from a large university hospital was assessed 4–8 weeks after discharge by a multidisciplinary team of rehabilitation professionals. Participants were between 29 and 71 days (mean 48 days) post-discharge from hospital; 32 participants required treatment in an intensive care unit (ICU group), and 68 were managed in hospital wards without needing ICU care (ward group). New illness-related fatigue was the most commonly reported symptom—by 72% of participants in the ICU group and 60.3% in the ward group. There was a clinically significant drop in EQ5D, of 68.8% in the ICU group and 45.6% in the ward group. The authors recommend planning rehabilitation services to manage post-discharge symptoms appropriately and maximize the functional return of COVID-19 survivors.
Simani et al. (Feb 2021) [34]	Research Article	The obtained data revealed the prevalence of CFS among patients with COVID-19, which is almost similar to CFS prevalence in the general population. Moreover, post-traumatic stress disorder (PTSD) in patients with COVID-19 is not associated with an increased risk of CFS. This study suggests that medical institutions should pay attention to the psychological consequences of the COVID-19 outbreak.
Townsend et al. (Feb 2021) [35]	Research Article	The results demonstrate the significant burden of fatigue, symptoms of autonomic dysfunction, and anxiety in the aftermath of COVID-19 infection but, reassuringly, do not demonstrate pathological findings on autonomic testing.
Graham et al. (Mar 2021) [36]	Research Article	A prospective study of the first 100 consecutive patients (50 SARS-CoV-2 laboratory-positive (SARS-CoV-2^+^) and 50 laboratory-negative (SARS-CoV-2^−^) individuals) presenting to the Neuro-Covid-19 clinic between May and November 2020 concluded that non-hospitalized COVID-19 “long-haulers” experience prominent and persistent “brain fog” and fatigue that affect their cognition and quality of life.
Toogood et al. (Mar 2021) [37]	Review	Viral infection is an established trigger for the onset of ME/CFS symptoms, raising the possibility of an increase in ME/CFS prevalence resulting from the ongoing COVID-19 pandemic.

**Table 2:** Main results of the survey of ME/CFS patients and undiagnosed persons.

Item	Persons’ Group	No. of Respondents	Mean	Standard Deviation (SD)	No. Responding “Yes”	%	95% Confidence Interval (%)
Age (Years)	Diagnosed	75	50.0	14.7			46.6–53.3
	Undiagnosed	222	45.1	12.9			43.4–46.8
Gender (No. Females)	Diagnosed	75			62	82.7	74.1–91.2
	Undiagnosed	226			187	82.7	77.8–87.7
Education (No. with Higher Education)	Diagnosed	74			32	43.2	32.0–54.5
	Undiagnosed	225			115	51.1	44.6–57.6
No. Living Alone	Diagnosed	74			25	33.8	23.0–44.6
	Undiagnosed	224			69	30.8	24.8–36.9
Household Income, per Member (EUR, p.a.)	Diagnosed	65	5364.4	2991.1			4637.3–6091.55
	Undiagnosed	213	6365.5	3819.7			5852.5–6878.5
No. Symptoms	Diagnosed	75	7.5	2.5			6.9–8.1
	Undiagnosed	231	6.3	2.8			5.9–6.7
Variability of Symptoms	Diagnosed	75			53	70.7	60.4–81.0
	Undiagnosed	231			178	77.1	71.6–82.5
No. Investigations	Diagnosed	75	5.7	3.2			5.0–6.4
	Undiagnosed	124	4.7	2.5			4.3–5.1
Out-of-Pocket Spending, to Mitigate Symptoms (EUR, p.a.)	Diagnosed	75	1143.0	125.1			1114.7–1171.3
	Undiagnosed	209	979.2	156.1			958.0–1000.4
Difficulty Explaining Illness to							
Physicians	Diagnosed	75			20	26.7	16.7–36.7
	Undiagnosed	231			76	32.9	26.8–39.0
Family	Diagnosed	75			35	46.7	35.4–58.0
	Undiagnosed	231			100	43.3	36.9–49.7
Friends	Diagnosed	75			20	26.7	16.7–36.7
	Undiagnosed	231			70	30.3	24.4–36.2
Employers	Diagnosed	75			30	40.0	28.9–51.1
	Undiagnosed	231			81	35.1	28.9–41.2
Quality of Life:							
Prior to Illness	Diagnosed	74	74.6	24.0			69.0–80.2
	Undiagnosed	212	74.1	22.0			71.1–77.1
In Past Year	Diagnosed	74	57.3	16.3			53.5–61.1
	Undiagnosed	219	58.1	16.8			55.9–60.3

## Data Availability

The aggregated data of the ME/CFS survey are available on request from the Riga Stradins University. The former COVID-19 patients’ survey data are available at the Latvian Biomedical Research and Study Centre.

## Supplementary Materials

The following are available online at https://www.mdpi.com/article/10.3390/jcm10143017/s1, Data S1: The patients’ survey on ME/CFS symptoms.
